# Macroalgae-Inspired Brominated Chalcones as Cosmetic Ingredients with the Potential to Target Skin Inflammaging

**DOI:** 10.3390/md23070278

**Published:** 2025-07-02

**Authors:** Ana Jesus, Sara Gimondi, Sónia A. Pinho, Helena Ferreira, Nuno M. Neves, Andreia Palmeira, Emília Sousa, Isabel F. Almeida, Maria T. Cruz, Honorina Cidade

**Affiliations:** 1 Associate Laboratory i4HB—Institute for Health and Bioeconomy, Faculty of Pharmacy, University of Porto, 4050-313 Porto, Portugal; 2 UCIBIO—Applied Molecular Biosciences Unit, Department of Drug Sciences, Faculty of Pharmacy, University of Porto, 4050-313 Porto, Portugal; 3 3B’s Research Group, I3BS—Research Institute on Biomaterials, Biodegradables and Biomimetics, University of Minho, AvePark, Parque de Ciência e Tecnologia, Rua Ave 1, Edificio 1 (Sede), Barco, 4805-694 Guimarães, Portugal; 4 ICVS/3B’s—PT Government Associate Laboratory, 4710-057 Braga, Portugal; 5 CNC—Center for Neuroscience and Cell Biology, Centre for Innovative Biomedicine and Biotechnology (CIBB), 3004-504 Coimbra, Portugal; 6 Laboratory of Organic and Pharmaceutical Chemistry, Department of Chemical Sciences, Faculty of Pharmacy, University of Porto, Rua de Jorge de Viterbo Ferreira, 288, 4050-313 Porto, Portugal; 7 CIIMAR/CIMAR LA—Interdisciplinary Centre of Marine and Environmental Research, University of Porto, Terminal de Cruzeiros do Porto de Leixões, 4450-208 Matosinhos, Portugal; 8 Faculty of Pharmacy, University of Coimbra, 3004-531 Coimbra, Portugal; 9 UNIPRO—Oral Pathology and Rehabilitation Research Unit, University Institute of Health Sciences (CESPU), 4585-116 Gandra, Portugal

**Keywords:** skin aging, macroalgae, brominated chalcones, anti-inflammatory activity, iNOS, Nrf2

## Abstract

Skin aging is mainly caused by external factors like sunlight, which triggers oxidative stress and chronic inflammation. Natural halogenated flavonoids have demonstrated anti-inflammatory properties. Inspired by the macroalgae-derived bromophenol **BDDE**, we investigated the anti-inflammatory potential of structure-related chalcones (**1**–**7**). Chalcones **1** and **7** showed the least cytotoxicity in keratinocyte and macrophage cells. Chalcones **1**, **2**, **4**, and **5** exhibited the most significant anti-inflammatory effects in murine macrophages after lipopolysaccharide stimulation, with chalcone **1** having the lowest IC_50_ value (≈0.58 μM). A SNAP assay confirmed that chalcones do not exert their effects through direct NO scavenging. Symmetrical bromine atoms and 3,4-dimethoxy groups on both aromatic rings improved the anti-inflammatory activity, indicating a relevant structure–activity relationship. Chalcones **1** and **2** were selected for study to clarify their mechanisms of action. At a concentration of 7.5 μM, chalcone **2** demonstrated a rapid and effective inhibitory action on the protein levels of inducible nitric oxide synthase (iNOS), while chalcone **1** exhibited a gradual inhibitory action. Moreover, chalcone **1** effectively activated the nuclear factor erythroid 2-related factor 2 (Nrf2) pathway with around a 3.5-fold increase at the end of 24 h at 7.5 μM, highlighting its potential as a modulator of oxidative stress responses. These findings place chalcone **1** as a promising candidate for skincare products targeting inflammation and skin aging.

## 1. Introduction

Skin aging manifests clinically through deep wrinkles, skin laxity, and hyperpigmented lesions, resulting from a complex interplay of intrinsic and extrinsic factors [1]. These changes are primarily driven by extrinsic factors, such as solar radiation, exposure to allergens, environmental pollutants, psychological stress, tobacco smoke, and dietary imbalances [2]. Collectively, these elements contribute to the increased production of reactive oxygen species (ROS) and reactive nitrogen species (RNS) [3]. Both ROS and RNS are involved in oxidative damage of skin cells and play key roles in activating the immune system and promoting inflammation [3]. The interplay between skin aging and inflammation is characterized by a bidirectional dynamic: chronic inflammation promotes oxidative stress and accelerates aging, while aging itself enhances inflammatory responses, a phenomenon commonly referred to as inflammaging [4,5]. Several molecular pathways can be targeted to counteract cutaneous inflammation and its deleterious effects, including (i) direct scavenging of ROS and RNS, thereby reducing oxidative damage; (ii) activation of the nuclear factor erythroid 2-related factor 2 (Nrf2) signaling pathway, which enhances the cellular antioxidant response and promotes cytoprotective gene expression; and (iii) modulation of inducible nitric oxide synthase (iNOS) expression, a major enzyme involved in inflammatory nitric oxide (NO) production [4,5]. Targeting skin inflammation through specific skincare products that incorporate novel anti-inflammaging agents holds promise for slowing the aging process, mitigating inflammation arising from external aggressions such as sun exposure and improving skin health [6]. In particular, targeting two key players, namely the Nrf2 pathway and the iNOS enzyme, represents a promising strategy to mitigate skin inflammaging. The Nrf2 pathway plays a pivotal role in cellular defense against oxidative stress by regulating the expression of antioxidant defenses, including superoxide dismutase (SOD), catalase (CAT), glutathione peroxidase (GSH), hemeoxygenase (HO-1), and NADPH quinone oxidoreductase 1 (NQO1), as well as cytoprotective genes, thereby contributing to the mitigation of inflammation through the suppression of oxidative stress and the inhibition of pro-inflammatory signaling pathways such as the nuclear factor kappa-light-chain-enhancer of activated B cells (NF-κB) pathway [7,8]. Upon its activation by external stimuli, Nrf2 translocates to the nucleus, where it binds to antioxidant response elements in the promoter regions of target genes. By enhancing the cellular antioxidant capacity, Nrf2 limits the accumulation of ROS, which are major amplifiers of pro-inflammatory signaling pathways, including NF-κB and MAPKs. Moreover, Nrf2 has been shown to directly suppress the transcriptional activity of NF-κB, thereby reducing the expression of pro-inflammatory cytokines [8]. Collectively, these mechanisms highlight the dual antioxidant and anti-inflammatory role of Nrf2 and support its therapeutic relevance in conditions characterized by chronic inflammation, including skin aging. In opposition, the iNOS enzyme contributes to the inflammatory environment by producing excessive nitric oxide, further promoting skin tissue damage [7,8]. Therefore, targeting both Nrf2 activation and iNOS inhibition offers a dual approach to counteracting the deleterious effects of inflammaging and to promote skin regeneration.

**Figure 1:**
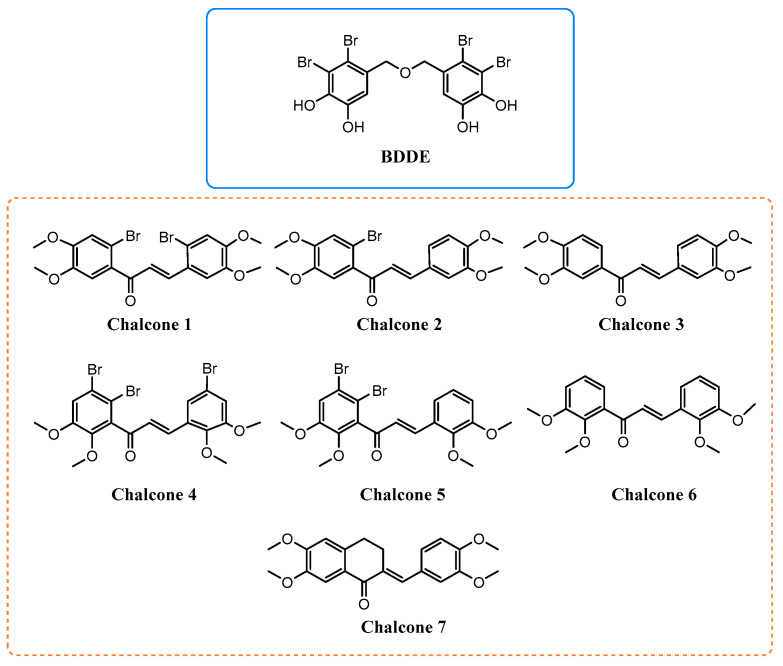
Chemical structure of the marine natural bromophenol **BDDE**, and the seven structure-related brominated and non-brominated chalcones (**1**–**7**).

Halogenated compounds, particularly chlorinated and brominated flavonoid derivatives, have been reported to exhibit potent anti-inflammatory activity [9,10,11,12]. Chalcones, a class of natural products distinguished by two benzene rings connected through a three-carbon α,β-unsaturated carbonyl system, have garnered significant attention. The biological activity of chalcones is shaped by the nature and position of their substituents, establishing the chalcone scaffold as a “privileged structure” in medicinal chemistry [13,14,15]. These flavonoids have demonstrated a wide range of biological properties, including anti-inflammatory effects mediated through various molecular pathways [16,17]. However, studies specifically exploring the anti-inflammatory potential of brominated chalcones remain limited, and their potential as anti-inflammaging agents, particularly in the context of photo-induced skin inflammation, has yet to be fully explored [18]. Beyond brominated chalcones, other brominated natural products have shown promising anti-inflammatory activity. One notable example is bis(2,3-dibromo-4,5-dihydroxybenzyl) ether (**BDDE**, Figure 1), a symmetric bromophenol produced by red and brown macroalgae [19]. Isolated from marine red and brown macroalgae species such as *Rhodomela confervoides* and *Leathesia nana*, **BDDE** has demonstrated a broad spectrum of biological activities, including anti-inflammatory effects [19,20,21]. Specifically, **BDDE** has been shown to reduce the production of inflammatory mediators, including NO, prostaglandin E2 (PGE2), and pro-inflammatory cytokines, such as tumor necrosis factor-α (TNF-α), interleukin (IL)-1β, and interleukin-6. It also inhibits the expression of iNOS in lipopolysaccharide (LPS)-stimulated murine macrophages [22]. Additionally, chalcone derivatives, namely dimethylamino-chalcones [23] and chalcones combined with methyl 2-hydroxyacetate moiety [24], have been already reported as demonstrating inhibitory action against the iNOS enzyme, through western blot analysis. BDDE-analogous chalcones (**1**–**7**, Figure 1), which retain the core structural motif of two benzene rings connected by a three-atom bridge, have previously been reported for their antibacterial and antifungal properties [25]. Building on the therapeutic potential of this group of compounds, we herein investigate the anti-inflammatory activity and mechanisms of action of a small library of BDDE-inspired brominated chalcones, previously synthesized and characterized by our team for their antimicrobial properties [25].

## 2. Results and Discussion

### 2.1. Cytotoxicity in Cell Lines

#### 2.1.1. Keratinocyte Cell Line

**Figure 2:**
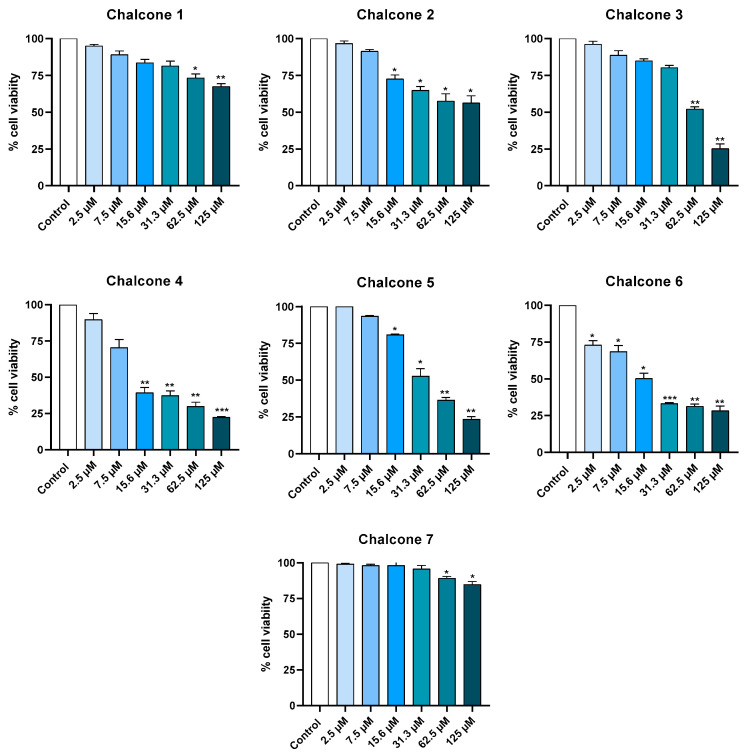
Cytotoxicity of chalcones **1**–**7** using a resazurin reduction assay in HaCaT cells by comparison with the control. Bar graphs represent the mean ± SEM of at least three independent experiments performed in triplicate. Statistical analysis: one ANOVA with Dunnett’s multiple comparisons test, where *p* < 0.05 was considered significant when compared to the control condition. * *p* < 0.05, ** *p* < 0.01, and *** *p* < 0.0005.

To evaluate the effects of chalcones **1**–**7** on cell viability after 24 h, a resazurin assay was performed using two cell lines: human HaCaT keratinocytes (HaCaT, CLS 300493), representing the epidermal layer, and murine RAW 264.7 macrophages (RAW 264.7, ATCC TIB-71), which model immune cells involved in dermal inflammatory responses. For the keratinocyte cell line, all seven brominated and non-brominated chalcones were tested across a concentration range from 2.5 μM to 125 μM (Figure 2). Chalcones bearing methoxy substituents at the 3,4-positions (**1**–**3** and **7**) did not exhibit cytotoxicity at the lower concentrations of 2.5 μM and 7.5 μM. Among these, chalcone **3**, without bromine atoms, resulted in a reduction superior to 50% in cell viability at the highest tested concentration of 62.5 and 125 μM. Among the chalcones containing 3,4-dimethoxy substitution, the tested concentration range allowed the determination of the IC_50_ value for chalcone **3**, which was 62.09 ± 1.07 μM.

In contrast, chalcones possessing methoxy groups at the 2,3-positions (**4**–**6**) demonstrated a more cytotoxic profile when compared with the chalcones with the 3,4-dimethoxy substitution pattern. At 2.5 μM and 7.5 μM, chalcones **4** and **5** showed no significant impact on keratinocytes viability. However, chalcone **6** led to approximately a 25% reduction in cellular viability at 2.5 μM. At the highest tested concentration (125 μM), all the chalcones with 2,3-dimethoxy substitution patterns (**4**–**6**) caused a substantial decrease in cell viability of around 75%. Among all the compounds tested, chalcones **1** and **7** exhibited the most favorable safety profile, showing statistically significant cytotoxic effects only at 62.5 μM and 125 μM, with a maximal reduction in viability not exceeding 25% and 15%, respectively. Furthermore, the tested concentration range enabled the determination of IC_50_ values for chalcones **4**–**6**, which were 11.73 ± 1.34 μM, 30.36 ± 2.66 μM, and 14.66 ± 3.97 μM, respectively. These results suggest that the position of methoxy substituents plays a more critical role in cytotoxicity than the degree of bromination, with 2,3-substitution enhancing a toxic effect to keratinocytes. Interestingly, bromination did not linearly correlate with toxicity. Overall, chalcones **1** and **7** emerged as the least cytotoxic compounds, making them safer, more promising candidates for further exploration for topical application.

#### 2.1.2. Macrophage Cell Line

**Figure 3:**
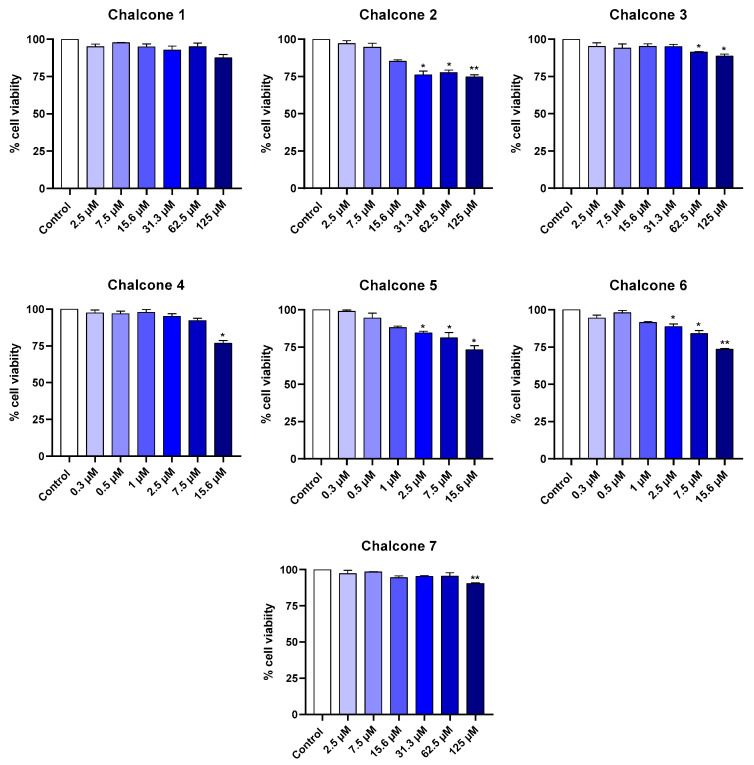
Cytotoxicity of chalcones **1**–**7** using the resazurin reduction assay in macrophages by comparison with the control. Bar graphs represent the mean ± SEM of at least three independent experiments performed in triplicate. Statistical analysis: one ANOVA with Dunnett’s multiple comparisons test, where *p* < 0.05 was considered significant when compared to the control condition. * *p* < 0.05, and ** *p* < 0.01.

To evaluate the cytotoxic effects of brominated and non-brominated chalcones **1**–**7** on macrophages, different concentration ranges were applied based on the response profiles previously observed for keratinocytes (Figure 3). Chalcone **1**, with a 3,4-dimethoxy substitution pattern and a symmetric bromination pattern, exhibited no cytotoxicity towards macrophages within the tested concentration range (2.5 to 125 μM). In turn, chalcones **2**, **3**, and **7** significantly reduced the cell viability of macrophage cells at the highest concentrations tested (62.5 and 125 μM) between 10 and 25%. Similarly, chalcones **4**–**6** demonstrated no significant toxicity in the lowest concentrations tested (0.3–1 μM) since macrophage viability remained above 80%. For this set of chalcones, a similar tendency was observed: a lower cytotoxic characteristic of chalcone **4**, which contained bromine atoms in both aromatic rings, compared to chalcones **5** (brominated in only the aromatic A ring) and **6** (non-brominated).

Overall, acceptable levels of cytotoxicity were observed up to 15.6 μM for chalcones **1**–**3** and **7**, up to 7.5 μM for chalcone **4**, and up to 1 μM for chalcones **5** and **6**. These results underscore the influence of methoxy and bromine substitution patterns on macrophage sensitivity. Chalcones **1** and **4**, brominated in both aromatic rings, demonstrated a safer profile in the range of concentrations tested comparatively with the chalcones of the respective set (**1**–**3** and **4**–**6**, respectively), suggesting that probably the presence of bromine atoms in only one aromatic ring or just dimethoxy substituents may exert greater cytotoxic effects. Regarding the influence of the dimethoxy substituents’ position, 2,3-dimethoxy derivatives (**4**–**6**) presented superior cytotoxicity compared to the 3,4-dimethoxy derivatives (**1**–**3** and **7**). Comparing the results of cytotoxicity studies in macrophage cells with the results in keratinocyte cells, a similar response was detected, with 2,3-dimethoxy derivatives showing superior cytotoxicity than the respective 3,4-dimethoxy derivatives. This may reflect differences in electronic distribution and steric hindrance that modulate cellular uptake. Moreover, the relatively mild cytotoxicity observed, particularly for 3,4-substituted chalcones (**1** and **7**), supports their potential for further investigation as anti-inflammatory agents, as they could offer a balance between bioactivity and cellular tolerance.

### 2.2. Anti-Inflammatory Activity

**Figure 4:**
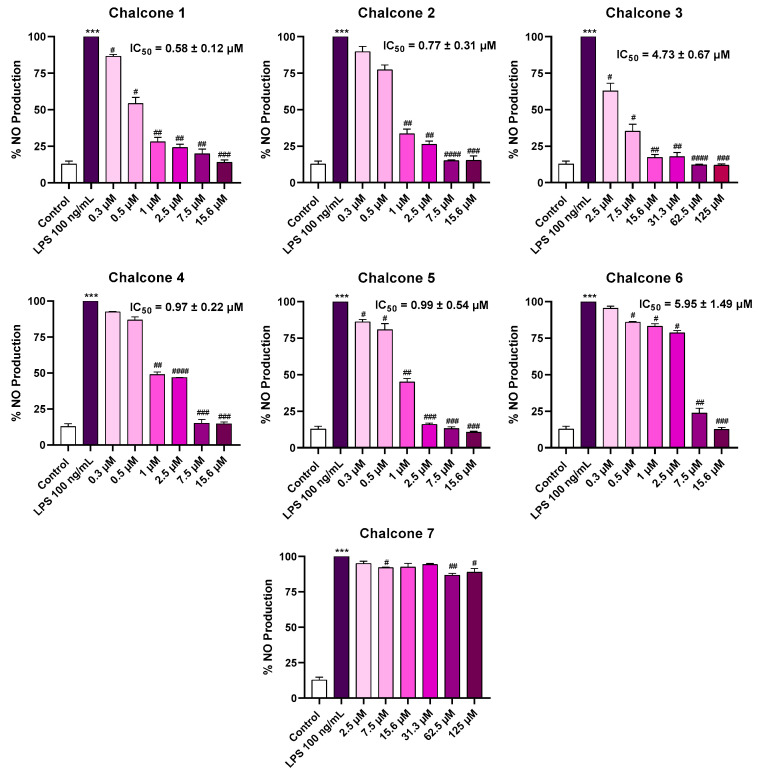
NO production by macrophages treated with LPS (100 ng/mL) or not (control) in the presence of chalcones **1**–**7**. Results are expressed as a percentage of NO production by cells treated with LPS (100 ng/mL). Each value represents the mean ± SEM of four independent experiments in duplicate. Statistical analysis: one ANOVA with Dunnett’s multiple comparisons test, where *p*< 0.05 was considered significant. *** *p* < 0.005, relative to the control, and ^#^ *p* < 0.05, ^##^ *p* < 0.01, ^###^ *p* < 0.0005, and ^####^ *p* < 0.0001, relative to LPS condition.

The anti-inflammatory potential of chalcones **1**–**7** was evaluated in macrophages cultured in the presence or absence (control) of LPS, as depicted in Figure 4. Under basal conditions (unstimulated, control), macrophages produce low levels of nitrites (≈12%). However, upon stimulation with LPS, a toll-like receptor 4 (TLR4) agonist, nitrite production significantly increases due to the upregulation of iNOS and consequent NO synthesis [26], as observed in the experiments. Each chalcone was tested at concentrations previously established as non-cytotoxic in macrophages, trying to ensure that observed changes in NO levels were not due to reduced cell viability. Among the seven chalcones, five chalcones (**1**, **2**, **4**–**6**) at the highest concentrations tested (15.6 μM) reduced the NO production to levels similar to the control, while chalcone **3** needed a concentration of 125 μM to exhibit this response. These results highlight the potential of these chalcones to abrogate LPS-induced NO production in macrophage cells. In turn, chalcone **7** only reduced by 12% the production of NO in LPS-stimulated cells, thus suggesting an inferior effect on the reduction of NO levels compared with the remaining chalcones (**1**–**6**). From Figure 4, it is also possible to conclude that none of the tested chalcones exhibited pro-inflammatory effects.

For six of the seven brominated and non-brominated chalcones evaluated (**1**–**6**), it was possible to determine the IC_50_ values. The lowest IC_50_ values were observed for the brominated chalcones (**1**, **2**, **4**, and **5**). Both chalcones **1** and **4**, which contain at least one bromo atom per aromatic ring, exhibited IC_50_ values of 0.58 ± 0.12 μM and 0.97 ± 0.22 μM, respectively, suggesting the importance of the presence of bromine atoms in both aromatic rings. Additionally, a symmetrical substitution pattern, as observed in chalcone **1**, allowed for obtaining a lower IC_50_ value (IC_50_ = 0.58 ± 0.12 μM) when compared with the non-symmetric substitution pattern of chalcone **4** (IC_50_ = 0.97 ± 0.22 μM). In contrast, chalcones **2** and **5**, which have bromo substitutes in only one aromatic ring (the one nearest to the ketone group present in the enone system—the aromatic A ring), exhibited IC_50_ values of 0.77 ± 0.31 μM and 0.99 ± 0.54 μM, respectively. These results support a structure–activity relationship (SAR) in which the symmetrical substitution pattern on both aromatic rings of chalcones may likely improve their biological efficacy. In fact, the presence of this type of substitution pattern results in good action towards inflammatory targets, namely the cyclooxygenase-2 (COX-2) [27,28] enzyme and the iNOS enzyme [29,30]. Studies on COX-2 inhibitors have shown that the presence of lipophilic groups, like a substituted phenyl ring or a bulky alkoxy substituent [28], as well as halogenate atoms [27], can improve the inhibitor’s binding affinity and selectivity [28].

Moreover, within this small library of chalcones, the position of the methoxy groups also appeared to influence the anti-inflammatory activity. Among the non-brominated chalcones, chalcone **3** (bearing methoxy groups at the 3,4-positions) was more effective (IC_50_ = 4.73 ± 0.67 μM) than chalcone **6** (with 2,3-methoxy substitution, IC_50_ = 5.95 ± 1.49 μM). A similar trend was observed among the brominated derivatives, where chalcones **1** and **2** (IC_50_ = 0.58 and 0.77 μM) outperformed their 2,3-methoxy analogues, chalcones **4** and **5** (IC_50_ = 0.97 and 0.99 μM), in terms of potency. This observation aligns with previous literature studies indicating that the positioning of the methoxy groups can modulate molecular interactions with inflammatory targets possibly by influencing hydrogen bonding capacity or steric hindrance at the active sites of enzymes, such as for the cyclooxygenase enzyme [31]. Two studies have already reported results that support this hypothesis for the iNOS enzyme [18,32], opening the pathway of the potential inhibitory action of these chalcones towards the expression and/or protein levels of the iNOS enzyme. Mechanistically, the observed reduction in NO levels may result from multiple actions of the chalcones, including (i) direct scavenging of reactive nitrogen species, (ii) inhibition of iNOS activity, or (iii) downregulation of iNOS expression at the transcriptional level by modulation of upstream signaling pathways such as NF-κB or MAPKs that govern the inflammatory response [33]. Building on these observations, we subsequently sought to elucidate the underlying mechanisms of action by in vitro and in silico studies.

### 2.3. NO Scavenging Assay (SNAP)

**Figure 5:**
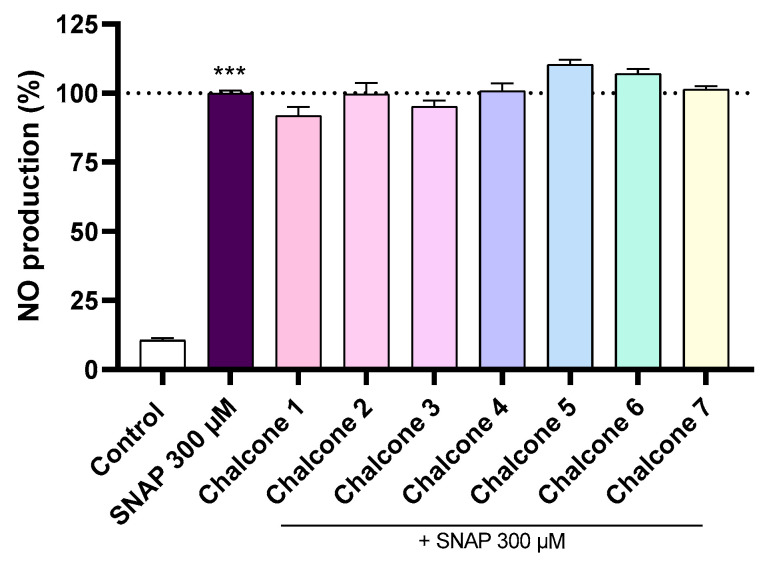
Scavenger activity of chalcones **1**–**7** at 7.5 μM in the presence of SNAP solution (300 µM). Bar graphs represent the mean ± SEM of at least three independent experiments performed in triplicate. Statistical analysis: one ANOVA with Dunnett’s multiple comparisons test, where *p* < 0.05 was considered significant when compared to the control condition. *** *p* < 0.0005.

The SNAP (*S*-nitroso-*N*-acetylpenicillamine) assay is a well-established method for assessing the NO scavenging capacity of compounds, offering valuable insights into their potential direct antioxidant and anti-inflammatory properties [34,35]. In this study, chalcones **1**–**7** were tested for their ability to capture NO radicals induced by the presence of SNAP at a concentration of 300 μM (Figure 5). The results suggest that neither brominated nor non-brominated chalcones displayed significant NO scavenging activity under these conditions. This finding suggests that their observed anti-inflammatory effects are unlikely to be mediated through direct NO neutralization. Instead, these compounds could potentially exert their effects via alternative mechanisms, such as the modulation of iNOS activity [33,34] or by targeting upstream targets in the inflammatory cascade, such as by the suppression of NF-κB activation [36], and/or Nrf2 activation [37], which will deplete co-activators from the iNOS enzyme, thereby reducing iNOS transcription and, consequently, iNOS protein levels and NO production [33].

### 2.4. iNOS Expression

To investigate the underlying mechanism of action of the most promising brominated chalcones (**1** and **2**), their effects on iNOS protein levels were assessed in LPS-stimulated macrophages at different time points (4, 6, and 24 h). The non-treated (NT) condition, consisting of macrophages stimulated with LPS but receiving no chalcone treatment, was used as the reference for maximal iNOS induction under inflammatory conditions and served as a positive control. The selected concentrations (7.5 μM and 15 μM) were selected based on chalcones **1** and **2** cytocompatibility (Figure 3) and efficacy in reducing NO production (Figure 4). Since NO production is a direct consequence of iNOS enzymatic activity, where the enzyme catalyzes the conversion of *L*-arginine to NO following inflammatory stimulation such as LPS, this provides a functional readout of iNOS expression and activity [33,38].

**Figure 6:**
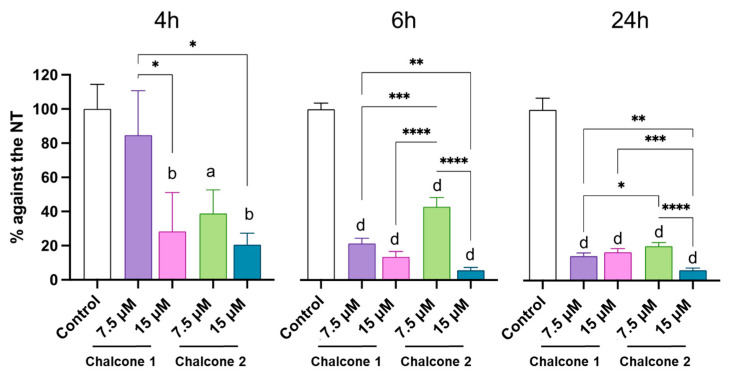
iNOS expression level after treatment of 4, 6, and 24 h with chalcones **1** and **2** in macrophage cell line. Results are expressed as volume arbitrary unit (A). Statistical analysis: Ordinary two-way ANOVA with Tukey’s multiple comparisons test where *p* < 0.05 was considered significant. * *p* < 0.05, ** *p* < 0.01, *** *p* < 0.005, and **** *p* < 0.001, relative to the LPS condition. Effect within each time point of brominated chalcones treatment over the iNOS expression levels (B). Results are expressed as a percentage normalized against the LPS condition. Statistical analysis: Ordinary one-way ANOVA with Tukey’s multiple comparisons test where *p* < 0.05 was considered significant. * *p* < 0.05, ** *p* < 0.01, *** *p* < 0.005, and **** *p* < 0.001, within conditions. Ordinary one-way ANOVA with Dunnett’s multiple comparisons test where *p* < 0.05 was considered significant: a = *p* < 0.05, b = *p* < 0.01, c = *p* < 0.005, and d = *p* < 0.001, related to the LPS condition (Control). NT: not treated.

Here, we focused on validating iNOS expression at the protein level by western blot (Figure 6). While gene-level expression was not assessed, it is well established that LPS stimulation induces both iNOS transcription and translation in RAW 264.7 macrophages [39,40,41]. Moreover, changes observed at the protein level are often closely associated with upstream transcriptional or translational modulation. Therefore, quantifying iNOS protein levels provides a biologically relevant measure of the chalcones’ inhibitory effects on this key inflammatory mediator. Indeed, the effects of chalcone **1** and chalcone **2** on iNOS expression revealed notable differences in both potency and time-dependent activity. At 7.5 µM, chalcone **1** showed a strong and time-dependent suppression of iNOS, with expression levels decreasing from 84.7% (±26) at 4 h to 14.3% (±2) at 24 h, suggesting a delayed but progressively stronger inhibitory effect. Increasing concentration to 15 µM enhanced this suppression at earlier time points, namely, 28.3% (±23) at 4 h, 13.5% (±3) at 6 h, and 16.7% (±2) at 24 h, indicating a plateau in effect from 6 h onward.

In contrast, chalcone **2** at 7.5 µM displayed a more pronounced reduction, with iNOS expression decreasing to 38.9% (±14) at 4 h and further halving by 24 h to 20.2% (±2). Interestingly, treatment with 15 µM chalcone **2** led to a much stronger suppression compared to the lower dose. At 4 h, the iNOS levels reached 20.5% (±7), which corresponds to the level achieved with 7.5 µM only after 24 h, suggesting that the higher concentration is approximately 6 times faster in reducing iNOS expression. At 6 h, iNOS levels dropped to 5.7% (±2), with no significant further reduction at 24 h 6.1% (±1), indicating an early onset of maximal inhibition.

Overall, the results demonstrate that chalcone **1** can reduce iNOS expression by up to approximately 85%, regardless of concentration. However, at 7.5 µM, the maximum suppression was only reached after 24 h, whereas at 15 µM, the peak effect was already evident at 6 h with no further reduction, indicating that the higher concentration leads to a 4 times faster inhibitory response. On the other hand, chalcone **2** exhibited more pronounced dose-dependent effects. At 7.5 µM, maximum iNOS suppression (~80%) was achieved at 24 h, similar to chalcone **1**. However, doubling the dose to 15 µM led to the same level of suppression already after 4 h, suggesting a 6 times faster inhibition. Moreover, at 15 µM, chalcone 2 showed a stronger inhibitory potential overall, reaching ~95% suppression at 6 h, a level that remained stable up to 24 h. These findings suggest that the reduction of NO levels is at least mediated through the inhibition of iNOS expression or fast interference with NF-κB pathway [29,33], particularly for chalcone **2**. For chalcone **1**, the gradual inhibitory profile, with its maximal efficacy observed at 24 h, suggests that its mechanism of action could be in the sustained downregulation of iNOS transcription and/or a cumulative antioxidant pathway activation (e.g., Nrf2 pathway), which requires longer periods to manifest [18,29].

### 2.5. Molecular Docking Studies

To gain deeper insights into the molecular interactions between the most promising chalcones (**1** and **2**) and the iNOS enzyme, molecular docking studies were conducted. Accurate modelling was ensured by using the experimentally determined crystal structure of iNOS, available in the Protein Data Bank. The active form of iNOS is a homodimer, with a single active site that contains a heme group, **H4B**, and a binding pocket [42,43]. A reference inhibitor was selected. Ethylisothiourea (**ITU**) is a well-characterized, substrate-like inhibitor that binds at the active site and blocks enzyme activity. This compound has been previously reported in association with the iNOS crystal structure [42,44]. In turn, the co-factor **H4B** (5,6,7,8-tetrahydrobiopterin) is essential for the iNOS enzyme. **H4B** ensures the dimerization of the two monomers, transforming the enzyme into its active form. Co-factor **H2B** (2-amino-6-(1,2-dihydroxypropyl)-7,8-dihydro-*6H*-pteridin-4-one), the oxidized form of **H4B**, could compete with **H4B**; however, the activation of the enzyme is only totally supported by **H4B** co-factor [42,43,44]. In this study, we employed two strategies to predict the action of chalcones towards the iNOS enzyme: (i) docking chalcones **1** and **2** into the catalytic site to assess their potential as competitive inhibitors, and (ii) evaluating their ability to displace or disrupt the co-factor **H4B** binding, thereby destabilizing the correct dimerization of the iNOS monomers, and thus inhibiting NO production.

In terms of docking scores (Table S1, Supplementary Materials), neither chalcone **1** nor **2** exhibited higher binding affinities than co-factor **H4B** of the iNOS enzyme. For the **H4B** co-factor, the chalcone scores were −7.2 kcal/mol (**1**) and −6.8 kcal/mol (**2**), and the control had a score value of −8.7 kcal/mol. Chalcones **1** and **2** exhibited better docking score values of −7.5 and −7.0 kcal/mol, respectively, when compared with the **ITU** inhibitor (−4.7 kcal/mol). Additionally, other studies [44,45] already reported docking score values for **ITU** similar to the obtained in this study (4.5–5.5 kcal/mol), allowing us to validate this study.

**Figure 7:**
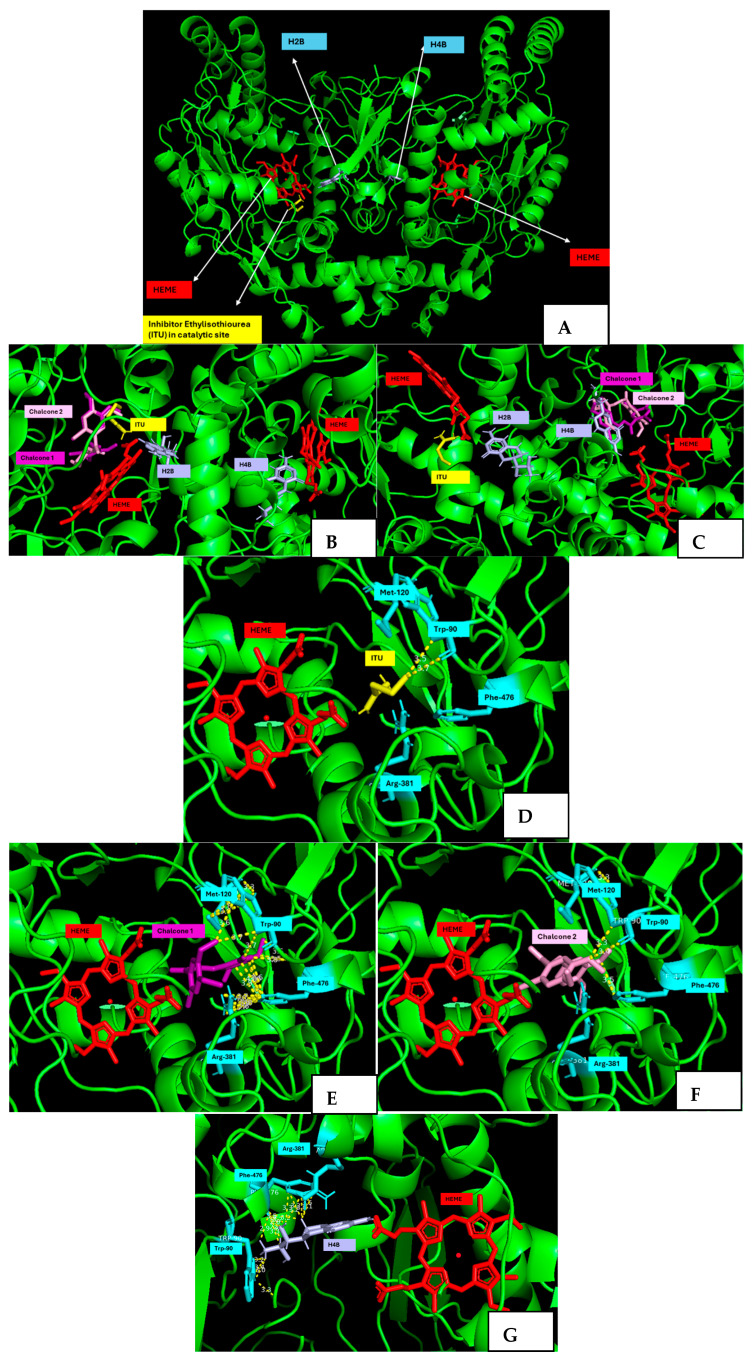

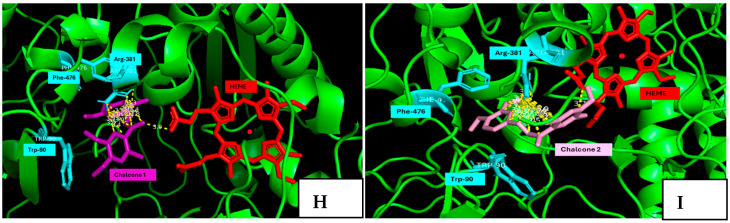
(**A**) Dimerized iNOS enzyme with two heme groups, two co-factors (**H4B** and **H2B**), and the well-known inhibitor **ITU**; (**B**) chalcones **1** and **2** and **ITU** represented in the catalytic site of the iNOS enzyme; (**C**) chalcones **1** and **2** and co-factor **H4B** in the local active site of the co-factor required for the dimerization and activation of the enzyme; (**D**–**F**) interaction between residues of the catalytic site with **ITU** inhibitor (**D**), chalcone **1** (**E**), and chalcone **2** (**F**); and (**G**–**I**) interaction between residues of the local active site of the co-factor **H4B** with the co-factor **H4B** (**G**), with chalcone **1** (**H**), and with chalcone **2** (**F**). Heme group: prosthetic group; **H4B** and **H2B** co-factors: 5,6,7,8-tetrahydrobiopterin and 2-amino-6-(1,2-dihydroxypropyl)-7,8-dihydro-6*H*-pteridin-4-one co-factors, and **ITU**: *S*-ethylisothiourea inhibitor. Phe: phenylalanine, Trp; tryptophan; Arg: Arginine; and Met: methionine.

Regarding amino acid residues, there are particularly important residues for the function of the catalytic active site. Arginine (Arg), methionine (Met), and glutamic acid (Glu) amino acid residues are essential for substrate recognition and binding to the iNOS enzyme. Other residues, such as tryptophan (Trp), and phenylalanine (Phe) are involved in interactions with **H4B** and contribute to the proper positioning of co-factor and substrate [42,43,44]. A visual inspection using PyMol allowed us to determine key molecular interactions (Figure 7) with the main amino acid residues identified for the catalytic active site (Met-120, Phe-476, Arg-361, and Trp-90) and for the local active site of the co-factor (Phe-476, Arg-388, and Trp-90).

At the catalytic active site of the iNOS enzyme, chalcones **1** and **2** interacted with Arg-381, Met-120, Trp-90, and Phe-476 amino acid residues. Chalcone **1** established more interactions than chalcone **2** with these residues. The aromatic B ring of chalcone **1** established π-π stacking and hydrophobic interactions (methoxy groups), with Met-120 and Trp-90, respectively. Additionally, bromine and methoxy groups from the aromatic B ring interacted with Arg-381 and Phe-476, mainly with their guanidino and indole chemical groups, respectively, possibly through hydrophobic, hydrogen interactions, and π-π stacking interactions. In turn, chalcone **2** only interacted through its methoxy groups, specifically the 4-OCH_3_ group of aromatic A and B rings, with Trp-90 and Phe-476 aminoacid residues. Compared with the **ITU** inhibitor, which only interacted with Trp-90 residue, chalcones **1** and **2** presented promising inhibitory profiles, which are associated with the more negative docking score values obtained of −7.5 and −7.0 Kcal/mol, respectively (Table S1, Supplementary Materials). These results from the catalytic site reinforce the information already reported by Fischman et al. and Crane et al., that larger competitive inhibitors will contribute to a better fitting in the catalytic pocket of the enzyme [42,44,45].

The interactions of chalcones **1** and **2** in the local site associated with the **H4B** co-factor were mainly π-π stacking between the aromatic A and B rings of the chalcones and the phenyl ring of Phe-476 and the imidazole ring of Trp-90 van der Waals and hydrogen bonding interactions with the aromatic B ring of the chalcones and their methoxy groups with the aromatic ring (Phe-476) and guanidino group (Arg-381), respectively. The α,β-unsaturated ketone moiety in both chalcones can participate in hydrogen bonding, with amino acid residues, such as Arg-381, capable of donating hydrogen bonds. In fact, these two compounds presented the lowest IC_50_ values for the NO production, once again highlighting the effect of 3,4-methoxy group substitution pattern on anti-inflammatory activity. Both interactions were observed that the distances between the active sites and the residues were inferior to 3.5 Å and 4.0 Å for chalcones **1** and **2**, respectively, possibly justifying the more negative score for the dibrominated chalcone (chalcone **1**: −7.2 kcal/mol) than for the monobrominated chalcone (chalcone **2**: −6.8 Kcal/mol). Additionally, chalcones **1** and **2** can establish a hydrogen interaction between α,β-unsaturated ketone group and the carboxylic acid end group present in the heme group, when compared to the **H4B** co-factor that cannot achieve that interaction.

Overall, the difference between the two compounds lies in the substitution pattern of the aromatic rings. Chalcone **1** has two identical 2-bromo-4,5-dimethoxyphenyl groups, which may enhance symmetrical interactions, while chalcone **2** has one 2-bromo-4,5-dimethoxyphenyl and one 3,4-dimethoxyphenyl group, which introduces some asymmetry and may alter/decrease the strength and orientation of the interactions at the catalytic active site and the **H4B** co-factor active site. Nevertheless, it is important to reflect that the in vitro studies were performed in mouse cells, while the docking studies used a human iNOS structure; thus, it will be important to further validate the docking results in human-derived cellular models, aiming to confirm the action of the chalcones.

### 2.6. Nrf2 Activation

**Figure 8:**
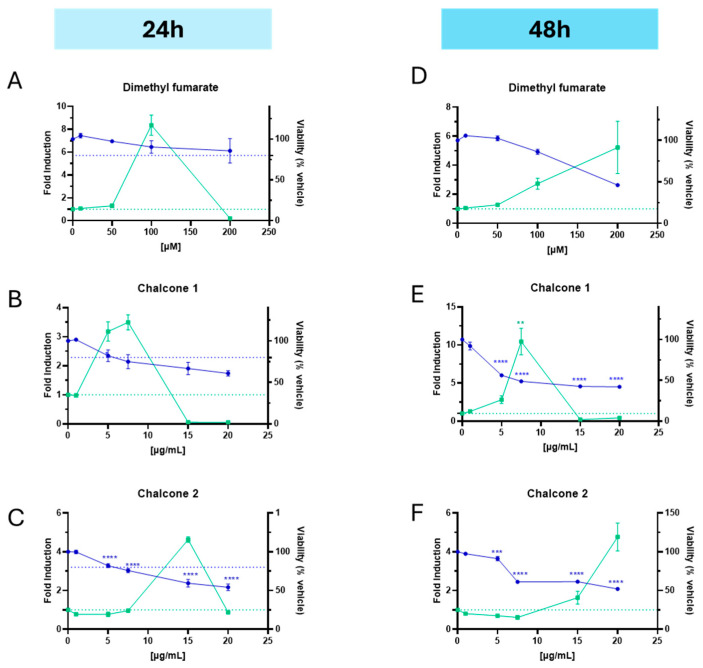
Nrf2 activation using the KeratinoSens™ assay in HaCaT cells transfected with an Nrf2 luciferase reporter construct, incubated for 24 h (**A**–**C**) or 48 h (**D**–**F**) with chalcones **1** and **2**. DMF was used as a positive control. Data represent the results from four independent experiments. Non-linear regression analysis was performed using GraphPad Prism v.9.0.0 for Windows. The blue line represents the results of cell viability (% regarding the control) and the green line represents fold induction. Statistical analysis: one ANOVA with Dunnett’s multiple comparisons test, where significance includes. * *p* < 0.05, ** *p* < 0.01, *** *p* < 0.0005, and **** *p* < 0.0001.

The KeratinoSens™ assay was originally developed to assess the skin sensitization potential of Nrf2 activation, but it has also been utilized to evaluate the ability of natural compounds to induce this transcription factor [46,47]. In this study, we assessed the capacity of chalcones **1** and **2** to activate Nrf2 after 24 and 48 h (Figure 8A–F). Chalcone **1** showed the most promising results by significantly activating Nrf2 at 24 h with concentrations of 5 µM and 7.5 µM, resulting in mean fold inductions of 3.18 and 3.49, respectively, compared to the vehicle (DMSO), without affecting cell viability (Figure 8B). The positive control, dimethyl fumarate (DMF), showed an Nrf2 activation with an 8.36-fold increase at 100 µM as expected [47]. However, at 48 h, Nrf2 activation was associated with a decrease in cell viability, suggesting potential cytotoxic effects at prolonged exposure times.

These findings indicate that chalcone **1** can activate the Nrf2 pathway in keratinocytes at non-cytotoxic concentrations within 24 h, highlighting its potential as a modulator of oxidative stress responses. The Nrf2 pathway plays a crucial role in cellular defense mechanisms by regulating the expression of antioxidant and phase II detoxification enzymes [7]. The Nrf2 is activated by Michael acceptors, electrophilic compounds with an α,β-unsaturated carbonyl group, such as the already market-approved drug DMF, and these chalcone derivatives may contribute to their anti-inflammatory properties by enhancing the cellular antioxidant capacity and suppressing pro-inflammatory signaling pathways, namely transcription factor NF-kB. However, the observed decrease in cell viability at 48 h underscores the importance of evaluating the temporal dynamics and concentration-dependent effects of Nrf2 activation to ensure therapeutic efficacy without inducing cytotoxicity.

## 3. Materials and Methods

### 3.1. Synthesis of Chalcones ***1**–**7***

**Scheme 1:**
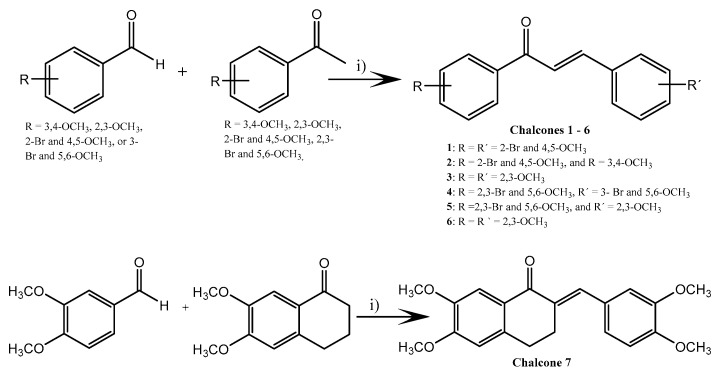
Synthesis of chalcones (**1**–**7**). (i) NaOH, MeOH, r.t., 2 h–1 week. Adapted from Jesus et al. [25].

The synthesis of brominated and non-brominated chalcones **1**–**7** (Figure 1, Scheme 1) was previously reported [25]. A solution of respective acetophenones (0.2–2.7 mmol) in methanol was prepared and put on ice. After, an aqueous solution of 40% sodium hydroxide was added until basic pH values of 13–14 was obtained. Then, a solution of appropriately substituted benzaldehyde (0.3–5.4 mmol) in methanol was slowly added to the reaction mixture. The reaction was left to stir for 2 h–1 week at room temperature and protected from the light. At the end, an appropriate amount of ice was added to the reaction mixture to quench it, and the pH adjusted to ≈5 with the addition of a diluted 1 M HCl solution. For the chalcones (**1**–**3**, **5**, and **7**), a solid was obtained, filtered, and washed with cold distilled water. Chalcones **3** (η = 74%) and **7** (η = 64%) were obtained without further purification. For chalcones **4** and **6**, the crude product (oil) followed a liquid–liquid extraction process with chloroform (3 × 25 mL) as the organic solvent. The organic phase was washed with a saturated solution of NaCl (2 × 20 mL), dried over anhydrous Na_2_SO_4_, and filtered, and the chloroform was evaporated under reduced pressure. Chalcone **1** (η = 87%) was purified by flash column chromatography (SiO_2_, n-hexane/ethyl acetate 98:2), followed by an extra purification step through TLC (SiO_2_, diethyl ether/petroleum ether 8:2). Chalcone **2** (η = 80%) was purified by TLC (SiO_2_, n-hexane/ethyl acetate 9:1). Chalcones **4** (η = 55%), **5** (η = 48%), and **6** (η = 85%) were purified by flash column chromatography (SiO_2_, n-hexane/ethyl acetate 95:5 and 9:1). The structure elucidation of all the BDDE-inspired chalcones was established by NMR and HRMS techniques and was in accordance with the data previously reported [25]. The presence of two doublets at δ_Hα_ 6.95–7.40 ppm and δ_Hβ_ 7.45–7.94 ppm with coupling constants between 15.5–16.4 Hz) confirmed the synthesis of the chalcones as well as their (*E*)-configuration.

### 3.2. Materials

Dulbecco’s Modified Eagle Medium (DMEM), sodium bicarbonate, sodium pyruvate, *L*-glutamine, *D*-glucose, trypsin-ethylenediamine tetra-acetic acid (EDTA) solution 1×, and resazurin sodium salt were purchased from Sigma-Aldrich (St. Louis, MO, USA). The fetal bovine serum (FBS) and penicillin–streptomycin were obtained from Gibco (Carlsbad, CA, USA). Dimethyl sulfoxide (DMSO) and LPS from *Escherichia coli* 026:B6 (dissolved in phosphate-buffered saline) were purchased from Sigma-Aldrich Co., St. Louis, MO, USA. The MitoSOX (M36008) red mitochondrial superoxide indicator was purchased from Invitrogen.

### 3.3. Cell Culture

Human keratinocytes (HaCaT, CLS 300493, Eppelheim, Germany) were cultured in DMEM supplemented with heat-inactivated FBS, and a penicillin and streptomycin antibiotic solution. The cell culture of keratinocytes was washed with 6 mL of phosphate buffer solution with 0.05% EDTA (Sigma-Aldrich, St. Louis, MO, USA) and detached using 3 mL of 0.05% trypsin + 0.02% EDTA in PBS solution when the cells reached 70–80% confluence in cell culture flasks. DMEM was also used to cultivate the mouse macrophage cell line (RAW 264.7, ATCC TIB-71, Manassas, VA, USA) together with 10% (*v*/*v*) non-inactivated FBS, 1.5 g/L sodium bicarbonate, and 100 U/mL penicillin and 100 μg/mL streptomycin stock (antibiotic solution). The macrophage culture was mechanically detached using a cell scraper, and the final amount of DMEM media with the cells was sub-cultured in a 1:10 ratio to the cellular suspension.

### 3.4. Cell Viability Assay

The resazurin reduction assay was employed to evaluate the metabolic activity of human epidermal keratinocyte (HaCaT) and murine macrophage (RAW264.7) cell lines, as outlined in a previous study [48]. In brief, HaCaT cells (1.0 × 10^5^ cells/mL) and RAW264.7 cells (5.0 × 10^4^ cells/mL) were seeded in 96-well plates (200 μL/well) and incubated overnight at 37 °C to allow for cell attachment. Following this, the cells were treated with different concentrations of the test compounds and kept at 37 °C for 24 h. After 20 h, resazurin (500 μM in sterile phosphate buffer solution, pH 7.4) was added to each well to achieve a final concentration of 50 μM, followed by an additional incubation of 4 h. Absorbance was then measured at wavelengths of 570 and 620 nm using a SpectraMax Plus 384 absorbance microplate spectrophotometer (Molecular Devices, San Jose, CA, USA). Each experiment was conducted in triplicate, with a minimum of three independent assays. The results were graphically presented as the percentage of cell viability versus chemical concentration, relative to the control.

### 3.5. Anti-Inflammatory Activity (NO Production)

The production of NO was evaluated by measuring nitrite accumulation in culture supernatants using the previously described Griess assay [49]. Cells were seeded at a concentration of 5.0 × 10^4^ cells/mL in a 96-well plate (200 μL/well) and allowed to stabilize overnight at 37 °C. Afterwards, the cells were treated with different concentrations of compound solutions and pre-incubated for 1 h at 37 °C. Subsequently, an LPS in PBS solution was added to each well, achieving a final concentration of 100 ng/mL, and the plate was left to incubate for 24 h. An amount of 150 μL of the cell culture supernatant was then transferred to a fresh 96-well plate, and an equal amount of Griess reagent [comprising a 1:1 mixture of 1% (*w*/*v*) sulphanilamide in 5% (*v*/*v*) phosphoric acid and 0.1% (*w*/*v*) naphthylethylenediaminedihydrochloride] was added. Following a 30 min incubation at room temperature in the dark, the absorbance was recorded at 550 nm using a Biotek Synergy HT plate reader (Biotek, CA, USA). The nitrite concentration in the supernatants was determined by comparing the absorbance of each sample to a sodium nitrite standard curve. The results, obtained from at least three independent experiments performed in duplicate, were expressed as a percentage of NO production by cells versus chemical concentration, relative to LPS condition (100%).

### 3.6. NO Scavenging Potential (SNAP Assay)

The ability to scavenge NO was assessed using S-nitroso-*N*-acetyl-D/L-penicillamine (SNAP) as a source of NO. The production of NO was quantified using the Griess reaction, following a previously described method [49]. The procedure consisted of incubating either the culture medium alone (control) or with the test compounds at a final concentration of 100 μM, along with 300 μM of SNAP, in 48-well plates for 3 h at 37 °C. Following this, equal amounts of the supernatants and Griess reagent were mixed and allowed to incubate in the dark for 30 min at room temperature. Absorbance at 550 nm was then measured using a Biotek Synergy HT plate reader (Biotek, CA, USA). The nitrite concentration in the supernatants was determined by comparing the absorbance of each sample to a sodium nitrite standard curve. All experiments were performed in triplicate.

### 3.7. Western Blot Analysis of iNOS Expression

The expression of the iNOS enzyme was analyzed by western blot. RAW264.7 macrophages were seeded at a density of 1 × 10^6^ cells/well in a 6-well plate and incubated for 24 h at 37 °C in a humidified atmosphere containing 5% CO_2_. After incubation, cells were treated with 7.5 µM or 15 µM of the most promising chalcones (**1** and **2**) for 30 min, followed by the addition of LPS to have a final concentration of 100 ng/mL. Cells were then incubated for 4, 6, or 24 h. At each time point, cells were washed with PBS and lysed in RIPA buffer. The lysates were collected in Eppendorf tubes and kept on ice, with intermittent vortexing to ensure thorough lysis. Samples were centrifuged at 18,000× *g* for 16 min at 4 °C, and the supernatant was collected. Total protein concentration was determined using the Pierce™ BCA Protein Assay Kit (Thermo Scientific, 23225, Waltham, MA, USA), following the manufacturer’s instructions. Equal amounts of protein (25 µg) were loaded onto Bolt™ 8% Bis-Tris Mini Protein Gels (Invitrogen™, NW00087BOX, Waltham, MA, USA) and separated via electrophoresis. Proteins were then transferred onto nitrocellulose membranes (Invitrogen™, IB24002) using the iBlot^®^ 2 dry transfer system (Invitrogen™, Waltham, MA, USA). After transfer, membranes were sectioned to separate the iNOS bands from those of the housekeeping protein GAPDH. Membranes were blocked for 1 h at room temperature in TBS-T containing 5% BSA and subsequently incubated overnight at 4 °C with primary antibodies against iNOS (Cell Signaling Technology, 16713120S, Danvers, MA, USA) or GAPDH (Abcam, ab181602, Cambridge, UK), diluted in TBS-T with 1% BSA. On the following day, membranes were washed three times with TBS-T and incubated for 1 h at room temperature with an HRP-conjugated secondary antibody (Cell Signaling Technology, 7074S) diluted in TBS-T with 1% BSA. After three additional washes, signal detection was performed using Clarity™ Western ECL Substrate (Bio-Rad, 1705061, Hercules, CA, USA), and chemiluminescent signals were acquired using the Odyssey FC imaging system (LI-COR, Lincoln, NE, USA). Densitometric analysis of iNOS bands was performed using Image Studio™ Software (version 5.2, LI-COR, Lincoln, NE, USA), with values normalized to the corresponding GAPDH bands.

### 3.8. Docking Studies

The three-dimensional crystal structure of human iNOS (PDB ID: 4NOS) was retrieved from the Protein Databank Bank. The structure was obtained by X-ray diffraction, and it has a resolution of 2.25 Å. For molecular docking purposes, the two domains of each co-factor and the catalytic site with the heme group were considered. The well-known reported inhibitor **ITU** (ethylisothiourea) and the co-factor **H4B** (5,6,7,8-tetrahydrobiopterin) were used as controls. **ITU**, **H4B**, and chalcones **1** and **2** were drawn using ChemDraw Professional 16.0 (Perkin Elmer Informatics, Waltham, MA, USA) and minimized using ArgusLab software (Mark Thompson and Planaria Software LLC, Richland, WA, USA) using the Austin Model 1 parameterization of the MNDO (AM1) method. Docking was carried out using AutoDockVina v1.1.2. (Scripps Research, La Jolla, CA, USA) [50]. The top nine poses for each catalytic site (L1 and L2) were collected for the controls and chalcones **1** and **2**, and the most favorable binding conformation was attributed to the lowest docking score value. The grid box size was set to 25 × 25 × 25 grid points, center coordinates were set for the catalytic site (−0.197 × 99.421 × 16.963) and for the co-factor **H4B** (8.979 × 97.662 × 12.266), exhaustiveness was set to 8, and docking considered the target as rigid and the ligands as flexible. Results were ranked based on their docking score (kcal/mol). For molecular visualization, PyMol v3.1 (Schrödinger, New York, NY, USA) was used [51].

### 3.9. Evaluation of the Nrf2 Activation

Selected compounds (chalcones **1** and **2**), and the positive control, DMF, were dissolved in sterile DMSO. Intermediate dilutions were prepared in culture medium, including the final concentration to be added to the 96-well plate, with a final volume of 200 µL per well. The KeratinoSens™ assay was conducted as previously described [46]. In brief, cells were seeded at a density of 10,000 cells per well in 96-well white opaque plates (Greiner Bio-One GmbH, cat. no. 655083, Kremsmünster, Austria) and incubated for 24 h. The medium was then replaced with fresh medium containing 1% FBS and the test compounds, and cells were incubated for an additional 24 and 48 h. DMF was used as the positive control since it is a known Nrf2 activator. Cell viability was assessed in the same cells using resazurin. After 20 or 44 h of exposure to the test compounds, 50 µM of resazurin solution (in sterile PBS) was added to each well, and the plates were incubated for 4 h at 37 °C, 5% CO_2_ Following the resazurin incubation, the supernatant was transferred to a transparent 96-well plate, and absorbance was measured at 570 and 620 nm using a Synergy HT multi-mode microplate reader (BioTek, Bad Friedrichshall, Germany). To measure luciferase activity, cells in the white opaque 96-well plates were washed with 1× PBS. An equal volume of 1× PBS and lysis buffer (One-GLO Luciferase, cat. no. E6130, Promega, Madison, WI, USA) was added to the wells, and the plates were incubated for 20 min at room temperature. Luminescence was then measured using a FLUOstar OMEGA Microplate Reader (BMG Labtech GmbH, Ortenberg, Germany).

### 3.10. Statistical Analysis

GraphPad Prism 8 for Windows (GraphPad Software, San Diego, CA, USA; www.graphpad.com, accessed on 21 January 2025) was used to perform the statistical analysis. Results were presented as mean ± standard error of the mean (SEM), originating from a minimum of three independent experiments in duplicate or triplicate. Statistical comparisons were accomplished using one-way ANOVA followed by Dunnett’s multiple comparison post-test. A *p*-value inferior to 0.05 was considered significant.

## 4. Conclusions

Skin aging is mainly driven by external factors, translated into an increase in oxidative stress and inflammation processes. Different pathways could be considered to target inflammation, including (i) via scavenging of reactive species, (ii) via the Nrf2 antioxidant pathway, and (iii) via iNOS enzyme expression. Brominated flavonoids, particularly chalcones, exhibit anti-inflammatory activity; however, their potential in the context of skin aging remains largely unexplored. Interestingly, **BDDE**, a compound derived from marine algae, exerts anti-inflammatory effects by inhibiting iNOS and other key inflammatory mediators. Building upon these findings, this study investigated the anti-inflammatory potential and underlying mechanisms of a small library of **BDDE**-inspired brominated and non-brominated chalcones (**1**–**7**), previously recognized for their antimicrobial activity.

This study demonstrated that brominated chalcones, particularly the symmetric dibrominated derivative **1**, hold substantial promise as anti-inflammaging compounds for mitigating oxidative stress and inflammation in skin cells. Building on the structural inspiration from the macroalgae-derived bromophenol **BDDE**, we identified some SAR correlations: bromine atoms in both aromatic rings, 3,4-methoxy substituents, and a symmetric substitution pattern enhance anti-inflammatory activity. The derivatives with 2,3-methoxy substituents correlate with increased cytotoxicity in keratinocytes when compared with 3,4-methoxy substituent derivatives. Chalcone **1** stands out as an interesting anti-inflammatory agent (IC_50_ ≈ 0.58 μM in the murine macrophage cell line), with dual modulatory effects through activation of the Nrf2 antioxidant pathway and the gradual suppression of iNOS protein expression levels at 7.5 μM. No direct NO scavenging action was observed for this compound.

These findings provide a promising approach to mitigate skin inflammaging by elucidating the active mechanisms underlying the anti-inflammatory profile of brominated chalcones. By correlating structural symmetry and halogenation with enhanced anti-inflammatory activity, this work advances the rational design of brominated chalcone derivatives. The selective modulation of both Nrf2 activation and iNOS inhibition supports a dual-pathway strategy aimed at combating oxidative stress and chronic inflammation associated with skin aging.

In the future, these compounds should be optimized to reduce their toxicity. It is important to note that previous studies have shown that molecular modification of the enone moiety, specifically the selective and total reduction of the α,β-unsaturated ketone moiety, resulted in compounds with lower cytotoxicity [25]. Therefore, such molecular modifications may be considered as a strategy to obtain less cytotoxic compounds. Additionally, based on predictive in silico studies (molecular docking), modifications to the enone moiety are unlikely to affect interactions with the most important amino acid residues when considering the local active site of **ITU**. However, if the local active site of the **H4B** co-factor, which is essential for the activation of the iNOS enzyme, is taken into account, alterations to the α,β-unsaturated enone moiety could influence the interactions with relevant amino acid residues. Future studies should also evaluate the efficacy of chalcone **1** in both in vitro and ex vivo human models of skin aging, focusing on its impact on downstream inflammatory mediators such as PGE2, IL-1β, and IL-6, to further substantiate its potential. Moreover, chalcone **1** warrants evaluation in pre-formulation studies and rational formulation development, with the goal of its incorporation into skincare products. This work highlights the potential of marine-inspired halogenated flavonoids as valuable candidates for future skincare formulations aimed at promoting skin health and delaying the skin aging process.

## Data Availability

Data are contained within the article or Supplementary Material.

## Supplementary Materials

The following supporting information can be downloaded at: https://www.mdpi.com/article/10.3390/md23070278/s1, Figure S1. Uncropped Western Blot images (*n* = 3). Chalcone **1**: Br2, chalcone **2**: R2, and NT: treated with LPS. Table S1. Score values (kcal/mol) for the local site of co-factor and the catalytic active site of iNOS enzyme for chalcones **1** and **2**.
