# Supplementary material for: Macroalgae-Inspired Brominated Chalcones as Cosmetic Ingredients with the Potential to Target Skin Inflammaging
Source: Mar Drugs. 2025 Jul 2;23(7):278. doi: 10.3390/md23070278 (PMC12299847; doi:10.3390/md23070278)
Supplement: Supplementary file 1 [file marinedrugs-23-00278-s001.zip › marinedrugs-3724723-supplementary.pdf]

marine drugs

Article

Macroalgae-inspired brominated chalcones as cosmetic ingredients with the potential to target skin inflammaging

Ana Jesus[1,2], Sara Gimondi[3,4], Sónia A. Pinho[5], Helena Ferreira[3,4], Nuno M. Neves[3,4], Andreia Palmeira[6,7], Emília Sousa[6,7], Isabel F. Almeida[1,2,*], Maria T. Cruz[5,8,*], Honorina Cidade[6,7,9]

[1] Associate Laboratory i4HB - Institute for Health and Bioeconomy, Faculty of Pharmacy, University of Porto, 4050-313 Porto, Portugal; anaaimjesus@gmail.com (A.J.);
[2] UCIBIO - Applied Molecular Biosciences Unit, Department of Drug Sciences, Faculty of Pharmacy, University of Porto, 4050-313 Porto, Portugal;
[3] 3B's Research Group, I3BS - Research Institute on Biomaterials, Biodegradables and Biomimetics, University of Minho, AvePark, Parque de Ciência e Tecnologia, Rua Ave 1, Edificio 1 (Sede), Barco, Guimarães 4805-694, Portugal; sara.gimondi@i3bs.uminho.pt (S.G.); helenaferreira@i3bs.uminho.pt (H.F.); nuno@i3bs.uminho.pt (N.M.N.);
[4] ICVS/3B's – PT Government Associate Laboratory, 4710-057 Braga, Portugal;
[5] CNC - Center for Neuroscience and Cell Biology and Centre for Innovative Biomedicine and Biotechnology (CIBB), 3004-504 Coimbra, Portugal; spinho@cnc.uc.pt (S.P.);
[6] Laboratory of Organic and Pharmaceutical Chemistry, Department of Chemical Sciences, Faculty of Pharmacy, University of Porto, Rua de Jorge de Viterbo Ferreira, 288, 4050-313 Porto, Portugal; apalmeira@ff.up.pt (A.P.); esousa@ff.up.pt (E.S.); hcidade@ff.up.pt (H.C.)
[7] CIIMAR/CIMAR LA - Interdisciplinary Centre of Marine and Environmental Research, University of Porto, Terminal de Cruzeiros do Porto de Leixões, 4450-208 Matosinhos, Portugal
[8] Faculty of Pharmacy, University of Coimbra, 3004-531 Coimbra, Portugal;
[9] UNIPRO - Oral Pathology and Rehabilitation Research Unit, University Institute of Health Sciences (CESPU), 4585-116 Gandra, Portugal;
* Corresponding authors: ifalmeida@ff.up.pt (I.F.A.) and trosete@ff.uc.pt (M.T.C.)

marine drugs

MDPI

Western Blot gels

Full-length, uncropped Western Blot (WB) images corresponding to the data presented in Figure 6 of the main manuscript. These blots represent iNOS expression levels in LPS-stimulated RAW 264.7 macrophages following treatment with the indicated chalcones at 4, 6, and 24 h.

WB n=1

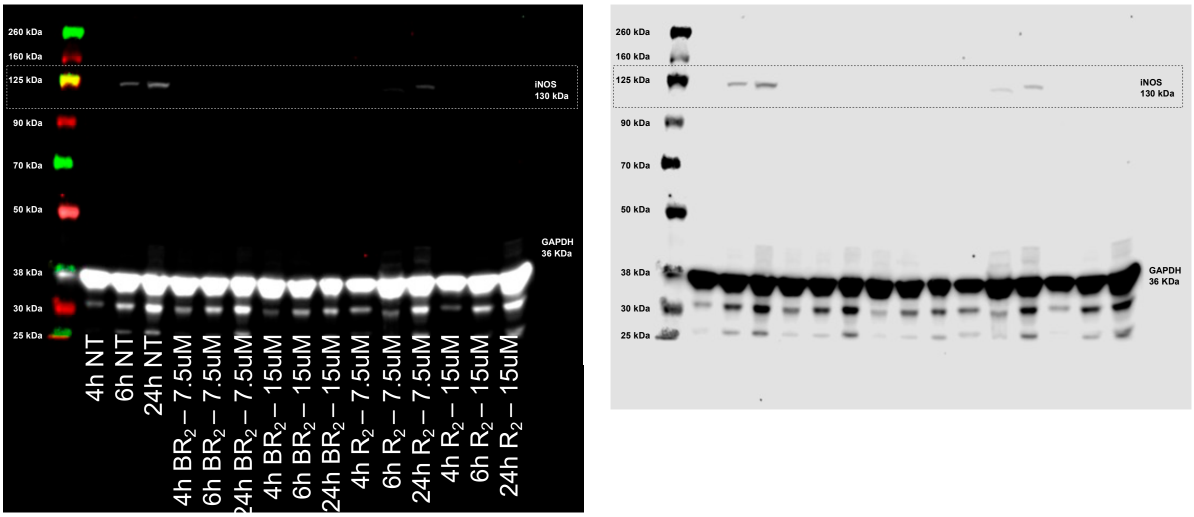

WB n=2

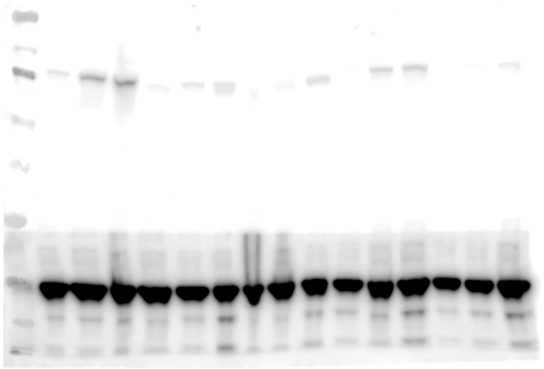

WB n=3

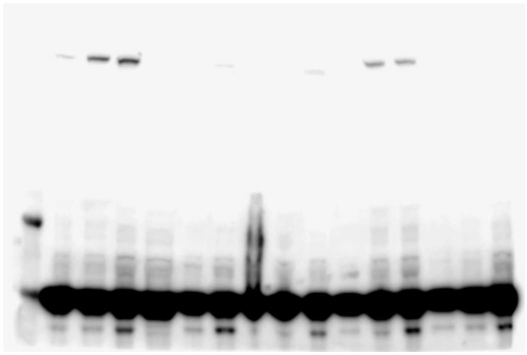

Figure S 1 Uncropped Western Blot images (n=3).Chalcone **1**: Br2, chalcone **2**: R2, and NT: treated with LPS.

Docking studies

Table S 1 Score values (kcal/mol) for the local site of co-factor and the catalytic active site of iNOS enzyme for chalcones **1** and **2**.

Compounds	Score values (kcal/mol)	
	Local site of co-factor	Catalytic active site
	H4B	ITU
**Chalcone 1**	- 7.2	- 7.5
**Chalcone 2**	- 6.8	- 7.0
**Control**	- 8.7	- 4.7
